# Stromal osseous metaplasia in urothelial carcinoma of the bladder: a rare case report and literature review

**DOI:** 10.1186/s13000-019-0851-z

**Published:** 2019-07-13

**Authors:** Joëlle Razafimahefa, Clément Gosset, Pierre Mongiat-Artus, Tsitohery Francine Andriamampionona, Jérôme Verine

**Affiliations:** 1 UPFR Anatomie et Cytologie Pathologiques, CHU, Andrainjato, Fianarantsoa, Madagascar; 2 Service de Pathologie, Hôpital Saint-Louis, AP-HP, Paris, France; 3 Service d’Urologie, Hôpital Saint-Louis, AP-HP, Paris, France; 4 Service de Recherches en Hémato-immunologie, CEA, Hôpital Saint-Louis, Paris, France; 5 Université Paris Diderot, Institut Saint – Louis, Paris, France

**Keywords:** Stromal osseous metaplasia, Metaplastic bone, Osteoid, Urothelial carcinoma, Bladder, Alkaline phosphatase

## Abstract

**Background:**

The bone formation within bladder tumors could be encountered in 3 conditions. These might consist of malignant bone formation in mesenchymal tumors; mixed mesenchymal and epithelial tumors; and epithelial tumors with stromal osseous metaplasia (SOM). This last is relatively rare. According to the English literature, only 12 cases have been reported in primary tumor and 7 in metastatic deposits of bladder primaries. Herein, we presented an additional case.

**Case presentation:**

An 83-year-old man was admitted 13 years ago for prostatic adenocarcinoma, treated with radical prostatectomy. Biochemical recurrence was detected 2 years after surgery (prostate-specific-antigen (PSA) level: 4.60 ng/mL) and progressively normalized (<1.0 ng/mL) after adjuvant radiotherapy and annual injection of leuprorelin (enantone^R^). He was referred after 8 years for hematuria, PSA level having slightly increased (0.60 ng/ml). Cystoscopy showed a nodular growth in the bladder wall, visualized as a calcified tumor on computed tomography (CT) and removed with transurethral resection. Histologically, the tumor consists of a non-muscle-invasive high grade papillary urothelial carcinoma with metaplastic bone within the stroma. Immunohistochemical analysis particularly demonstrated positive expression of respectively CD56 on osteoblasts, and CD68 on osteoclasts. MDM2 and CDK4 were negatives on osteoid and bone tissue. Six courses of Bacillus Calmette-Guerin (BCG) therapy have been administered. Two local recidives have occurred during an 8-month follow-up period after immunotherapy and were treated with six further courses of BCG therapy. At one-month follow-up, the patient was well without remaining symptoms.

**Conclusion:**

SOM is a rare benign condition whose pathogenesis remains uncompletely defined. Sarcomatoïd carcinoma represents the main differential diagnosis that influences therapeutic procedures. Prognosis depends essentially on the extent of the carcinomatous component .

## Background

The bone formation within bladder tumors, isolated or associated with cartilage formation, can be schematically divided into 3 distinct groups [1–4]. The first group consists of malignant bone formation in mesenchymal tumors (osteosarcoma). The second group corresponds to sarcomatoid urothelial carcinomas where the bone formation is considered as an heterotopic sarcomatous element associated with a carcinomatous component [1, 4]. In the third group, the bone formation appears as a stromal osseous metaplasia, that is, the transformation of a part of the tumoral stroma of urothelial carcinomas into mature bone, characterized by reactive benign bone trabeculae forming a lamellar architecture [3]. This last condition is rarely encountered in bladder tumors and represents a curious finding, firstly described in urothelial carcinoma by Pang et al. in 1958 [1]. Since then, a total of 19 cases have been reported in urothelial carcinoma among which 12 cases were found in primary tumors and 7 in metastatic locations [2].

We reported herein an additional case of stromal osseous metaplasia in a high grade papillary urothelial carcinoma of the urinary bladder.

## Case presentation

**Fig. 1:**
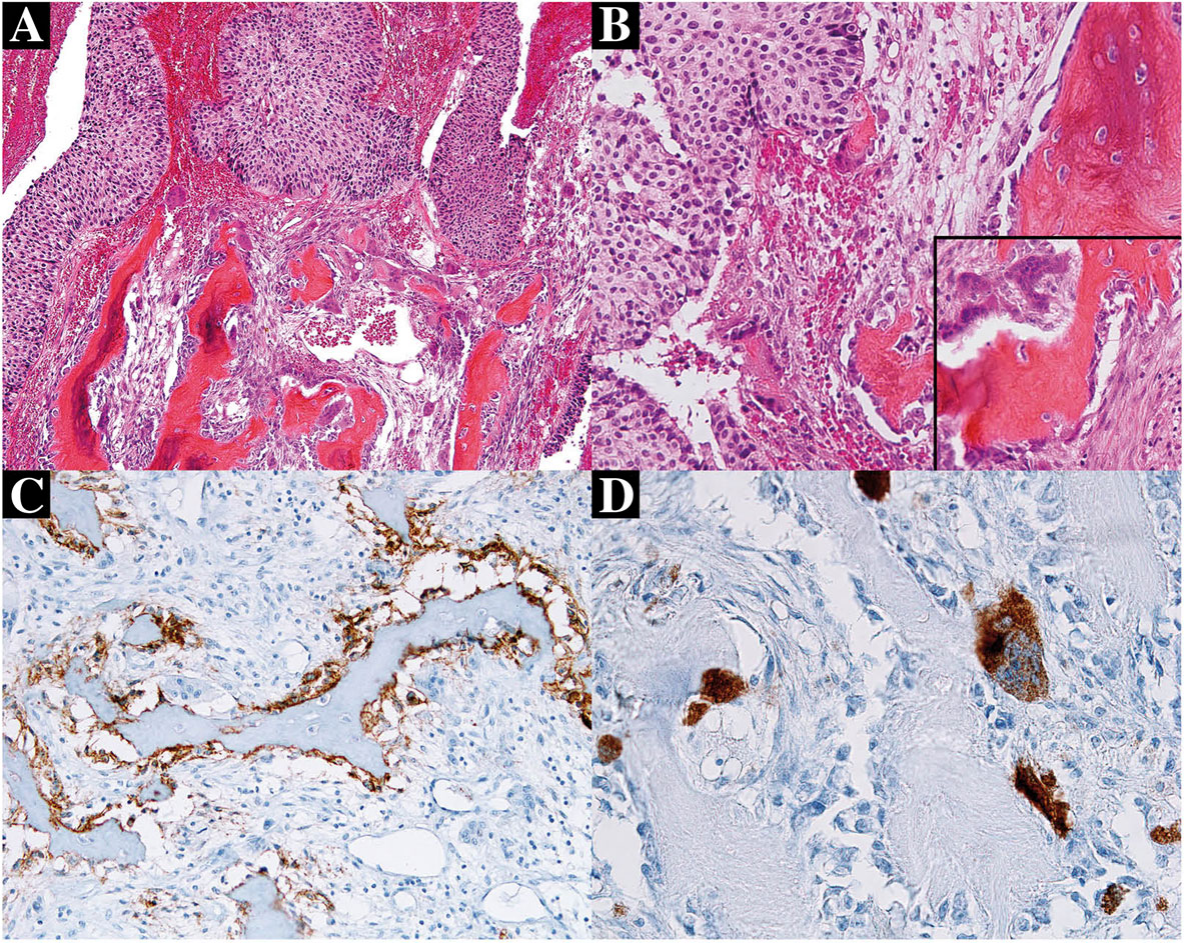
Stromal osseous metaplasia in urothelial carcinoma of the bladder. **a** Tumor stroma containing multiple bone trabeculae of variable size, separated by loose fibrous connective tissue with osteoid seams (Hematoxylin and Eosin Stain × 200). **b** Osteoid and bone trabeculae outlined by large cells with abundant cytoplasm and multinucleated giant cells, corresponding respectively to osteoblasts and osteoclasts. Transitional cell carcinomatous component is seen on the left. (Hematoxylin and Eosin Stain × 400). **c** Positive and diffuse membrane staining of osteoblasts with CD56 antibody. **d** Strong positive cytoplasmic staining pattern of osteoclasts with CD68 antibody

An 83-year-old man was firstly admitted to hospital 13 years ago for prostatic adenocarcinoma and underwent radical prostatectomy. He also received adjuvant radiotherapy and annual injection of leuprorelin (enantone^R^) for a biochemical recurrence characterized by a prostate-specific-antigen (PSA) level of 4.60 ng/mL detected 2 years after surgery. Clinical evolution was favorable and serum PSA level has progressively decreased to lesser than 0.1 ng/mL. At 8-years follow-up, he presented with an abundant gross hematuria. Serum PSA level was slightly increased (0.60 ng/ml). A cystoscopy was performed showing a nodular tumor growth on the right side of the lateral bladder wall that appeared as a large calcified tumor on computed tomography. Transurethral resection of bladder (TURB) was carried out. Tumor specimens consisted of small fragments of whitish firmer tissue. Microscopic examination showed frond like papillary projections which were lined by thick layers of stratified transitional epithelial cells with central fibrovascular cores. There was a predominant disorderly pattern with marked variation of architectural features. Tumor epithelial cells focally showed prominent cytologic abnormalities with moderate to significant nuclear pleomorphism. Quite numerous mitotic figures were observed and prominent nucleoli were also present in places. In some areas, the tumor cells disrupted the basement membrane and invaded into the lamina propria. The muscularis propria was respected. All these findings were consistent with the diagnosis of high-grade papillary urothelial carcinoma with infiltration of lamina propria (pT1 stage). Coexisting urothelial carcinoma in situ was found focally (Cis). The tumor stroma contained multiple bone trabeculae of variable size, lamellated or coarsed-fibred and separated by loose fibrous connective tissue with osteoid seams (Fig. 1a). Those osteoid and bony structures were outlined by numerous isolated large cells with abundant blue cytoplasm and quite numerous multinucleated giant cells that respectively correspond to osteoblasts and osteoclasts, without atypia nor pleomorphic stromal cells (Fig. 1b). No cartilage formation was observed. Immunohistochemistry showed in osteoid areas and bone tissue, a positive expression of CD56 (Fig. 1c) in osteoblasts. There was a discrete and heterogeneous staining for PS100, a discrete immunoreactivity for P53, and a Ki67 index of 20% in tumor cells. MDM2 and CDK4 immunostaining was negative in both metaplastic bone and carcinomatous components. Multinucleated osteoclasts were also strongly stained by CD68 (Fig. 1d). At 2-months follow-up after TURB, a control cystoscopy was performed and showed necrotic zone and retractile fibrous scar on the right side of the bladder trigone which were completely excised by secondary TURB. Those remaining lesions correspond essentially to fibro-inflammatory changes without residual tumor. Six courses of BCG therapy have been indicated. Two local recidives with recurrent episodes of gross hematuria have occurred during an 8-month follow-up period after immunotherapy. These local tumor recurrences displayed the same histological findings that demonstrated a high grade papillary urothelial carcinoma with superficial invasion of the lamina propria but were devoided of SOM foci. The multidisciplinary discussion recommended six further courses of BCG therapy at the time of the first recurrence. The second tumor recurrence appeared 2 months later and has been completely excised.

At one-month follow-up, the patient was well and did not complain of any symptoms.

## Discussion

Stromal osseous metaplasia is an unusual entity, rarely encountered in urothelial bladder carcinomas. There are 19 reports of such cases recorded to date in the English literature. These are summarized with the present case in Table 1 and Table 2. Patients are predominantly males (male to female ratio: 3.25) with a median age of 65 years (range 3 to 85). There was a unique pediatric case observed in a 3-year-old boy [5].

**Table 1:** Reported cases of SOM in papillary urothelial carcinoma of the bladder

Case	Age-Sex	Tumor site, description ± related circumstances	Histological findings	IHC profile within the metaplastic component	Treatment	Pathologic stage and grade at the diagnosis	Outcome	Reference
1	60y-F	Almost entire bladder: firm and irregular ulcerated mass, extremely fixated to the bladder wall with areas of calcification, 6y. after BTR and repeated cystodiathermy cauterization for bladder tumor.	-WD PUC -SOM characterized by thick lamellated mature bone trabeculae, outlined by osteocytes and surrounded by osteoid material. -Other findings: squamous metaplastic changes, calcified material, no cartilage formation.	WNP	Partial cystectomy.	pT2G3	NA	[1]
2	50y-M	Anterior, posterior and lateral bladder wall: multiple, nodular and partially pedunculated growths, 4y. after BTR and various occasions of cystodiathermy cauterization.	-Papillary urothelial tumor with doubtful stromal invasion. -SOM characterized by osteoid and multiple slender bone trabeculae, lamellated or coarse – fibred, outlined by active osteoblasts and small osteoclasts. - Other finding: no cartilage formation.	WNP	Cystodiathermy cauterization after biopsy.	p TxG1	Free from lesions and symptoms 5y after biopsy.	[1]
3	68y-M	Bladder, NOS.	-WD PUC -SOM: osteoid material and bone trabeculae, NOS -Other finding: no cartilage formation.	NA	NA	p TxG2	NA	[2]; [4]
4	65y-M	Right and left sides of the bladder wall: respectively large infiltrating solid tumor and papillary growth pattern.	Right side solid tumor: -PD urothelial carcinoma. -SOM: osteoid formation without mineralization. -Other findings: squamous metaplastic changes; focal calcification; no cartilage formation. Left side papillary tumor: -WD PUC	WNP	Radiotherapy after biopsies.	p T1G2	Died of pyelonephritis.	[4]
5	56y-M	Hard solid bladder tumor, NOS.	-WD PUC -SOM: several bone trabeculae mostly calcified without visible osteoblasts. -Other findings: bone marrow- like spaces containing fat, reticulum and capillaries, without hematopoietic elements; squamous metaplastic changes, no cartilage formation	WNP	Left the hospital without further treatment after biopsy.	p TxG2	NA	[4]
6	66y-M	Superior to the left ureteral orifice and posterior bladder wall: papillary stalked tumors, 18 m. after partial cystectomy and right ureteroneocystostomy for papillary urothelial carcinoma.	-MD PUC -SOM: osteoid formation with fibroblasts and osteoblasts in surrounding stroma. -Other finding: no cartilage formation.	WNP	Total cystectomy with pelvic lymphadenectomy and ureterosigmoidostomy.	p TxG2	Alive and well after surgery.	[6]
7	85y-F	Retrotrigonal region: solitary and pedunculated bladder tumor.	-PD PUC -SOM: osteoid material and bone trabeculae, NOS. -Other finding: cartilage formation.	WNP	-Repeated transurethral BTR. -Total cystectomy and ileal conduit diversion.	p T2b G3	NA	[7]
8	84y-M	Bladder neck: large exophytic tumor; 4y. after retropubic prostatectomy for prostatic hyperplasia, 5y. history of bladder urothelial carcinomas with TUBR and a 6-week course of intravesical thio-tepa.	-PUC with extensive glandular differentiation. -SOM: scattered foci of osteoid, often surrounded by flattened cells resembling osteoblasts. -Other findings: focal calcification, no cartilage formation.	WNP	TUR.	p T2 G3	Alive with disease 7 m. after last tumor resection.	[2]
9	60y-M	Posterior bladder wall: ill-defined sessile mass with hemorrhagic ulceration; 5 m. after TUR-prostate for benign prostate hyperplasia on which a tumor was seen and interpreted as NV-UC.	-NV- UC: small nests and cords of uniform cells with occasional tubular structures. -SOM: mature and laminated bone trabeculae with benign osteocytes.	WNP on metaplastic components.	Radical cystoprostatectomy with adjuvant chemotherapy.	p T4a Nx Mx	Died of metastatic disease 11 m. after the first diagnosis.	[8]
10	64y-M	Lateral and posterior bladder wall: multiple nodular lesions; 26y. history of bladder amyloidosis treated by repeated TUR (6 times, last 7y. ago).	-SUC with sarcomatoid differentiation and amyloidosis -SOM, NOS	WNP	Radical cystectomy with ileal conduit and adjuvant chemotherapy.	p T3b G3 N1 M0	Alive and well.	[9]
11	66y-M	Posterior bladder wall: firm white – tan mass with an extensive area of ossification near the right seminal vesicle.	-PUC, NOS -SOM: mature lamellated bone trabeculae outlined by osteoblasts.	WNP	-Radical cystoprostatectomy. -Chemotherapy (weekly taxane injection). -External beam radiotherapy.	p T4b G3	NA	[10]
12	3y-M	Posterior bladder wall: exophytic papillomatous tumor with a thin stalk.	-PUC -SOM, NOS -Other findings: glandular and squamous metaplasia.	WNP on metaplastic bone.	-TUR.	p T1 G3	Free from lesions at 2y. follow-up.	[5]
13	83y-M	Right side of the bladder wall: whitish and firmer nodular growth, 13y. after RP, adjuvant radiotherapy and annual injection of leuprorelin (enantone^R^) for prostatic adenocarcinoma.	-WD PUC with focal component of urothelial carcinoma in situ. -SOM: multiple bone trabeculae, lamellated or coarsed-fibred with osteoid seams outlined by osteoblasts and osteoclasts. -Other finding: no cartilage formation.	-CD56+ on osteoblasts. -CD68+ on osteoclasts. -Mild positivity of PS100 and P53 in osteoid areas and bone trabeculae. -MDM2-, CDK4-. -Ki 67 + 20%.	TUBR with adjuvant BCG therapy.	p T1 G3	-Two local recurrences over an 8-month follow-up period. -Alive and well at the time of the report.	Present case

*IHC* Immunohistochemical, *NA* not available, *NOS* not otherwise specified, *WNP* was not performed, *SOM* stromal osseous metaplasia, *y* years, *m* month, *PUC* papillary urothelial carcinoma, *SUC* solid urothelial carcinoma, *NV-UC* nested variant urothelial carcinoma, *PD* poorly differentiated, *MD* moderately differentiated, *WD* well differentiated, *BTR* bladder tumor resection, *TUR* transurethral resection, *TUBR* transurethral bladder resections, *RP* radical prostatectomy

**Table 2:** Reported cases of SOM in metastatic papillary urothelial carcinoma of the bladder

Case	Age-Sex	Tumor site, description ± related circumstances	Histological findings	IHC profile within the metaplastic component	Treatment	Grade	Outcome	Reference
1	54y-M	Abdominal wall, midway between umbilicus and pubis symphisis: hard fibrous plaque of 2,2 × 1,6 × 1 cm with central bone formation, 4y. history of papillary urothelial bladder carcinoma with prostatic enlargement treated by transurethral bladder resections, cystodiathermy cauterizations and transvesical prostatectomy.	-Well differentiated papillary urothelial carcinoma surrounded by fibrous connective tissue, presumed as scar tissue; and muscle. -SOM characterized by well-formed bone.	WNP	Surgical resection.	1	NA	[11]
2	65y-M	Left supraclavicular region: firm and partially fixed mass, measuring 1,5 × 1,5 cm, 2y. after radiotherapy and partial cystectomy for a papillary urothelial bladder carcinoma.	Left supraclavicular region: fibro-adipose tissue with prominent metaplastic bone formation and several foci of malignant cells consistent with metastatic papillary urothelial carcinoma.	WNP	Irradiation.	3	NA	[12]
		Left tibia: extensive lysis and sclerosis of the entire tibial shaft, 1y. after supraclavicular metastatic localization.	Left tibia: poorly differentiated papillary urothelial carcinoma invading bone, soft tissue and striated muscle with metaplastic bone formation.					
		Left femur proximal end: third metastatic focus, 8 m. after tibial metastasis.	Left femur proximal end: similar histological pattern to supraclavicular and tibial metastases.					
3	54y-M	Left tibia: metastatic localization, 14 m. after primary tumor.	NA	NA	NA	NA	NA	[13]
4	74y-F	Left tibia: metastatic localization with amorphous ossification in surrounding soft tissue, 14 m. after primary tumor.	Metastatic papillary urothelial carcinoma with new bone.	NA	NA	NA	NA	[13]
5	64y-F	Right femur, right and left tibiae: metastatic tumor with ill-defined bone destruction and heterotopic soft tissue ossification, 24 m. after primary tumor.	NA	NA	NA	NA	NA	[13]
6	78y-M	Massive nodal metastases on radical cysto-prostatectomy with lymph node dissection specimens for urothelial bladder tumor.	Metastatic papillary urothelial carcinoma with osseous metaplasia seen only in lymph nodes.	NA	Radical cystoprostatectomy with lymph node dissection.	3	NA	[14]
7	75y-M	. Nodal metastases on radical cysto-prostatectomy with bilateral ilio-obturator lymph node dissection specimens for urothelial bladder tumor. -Right retroperitoneal space: heterotopic bone formation, 1y. after surgery.	-Nodal metastases: poorly differentiated papillary urothelial carcinoma with small foci of mature lamellar-bone trabeculae, outlined by non atypical osteocytes. -Right retroperitoneal space: Similar histological pattern to nodal metastases.	WNP	Adjuvant chemotherapy and radiotherapy.	3	-Alive with progressing lesions resistant to radiotherapy at the time of the report. -No further available follow-up.	[3]

Among these cases, 12 occurred in primary tumor without predilection for any particular site within the bladder. In almost half of those cases, a past history of partial cystectomy or radical prostatectomy have been noticed 5 months to 7 years before, eventually associated with repeated procedures of TURB. For the remaining 6 cases, there was no data available describing circumstances related to the primary tumor. In our case, SOM was detected in a primary bladder tumor that occurred 13 years after a prostatic adenocarcinoma, treated by radical prostatectomy with adjuvant radiotherapy, followed by annual injection of leuprorelin (enantone^R^).

Moreover, apart from those 12 previous cases of primary urothelial carcinoma, 4 other cases of bladder carcinoma including one squamous, two undifferentiated and one adenocarcinoma have been reported by Eble and Young [2] in 1991 as associated with SOM. Besides, it is noteworthy that moderate to significant mucin deposits have been observed within the two latter variants.

Osseous metaplasia has been also found associated with metastatic tumors in 7 cases. Bone represents the most common site of metastasis and tibia is mainly involved associated as well with femur involvement [12, 13]. In such cases, radiographic evidence of new bone deposits has been noticed and heterotopic ossification has been described in soft tissue adjacent to the metastatic urothelial carcinoma. Other localizations including regional lymph nodes [3, 14], supraclavicular region [12], retroperitoneal space [3] and abdominal wall [11] have been also encountered.

Although the prognosis is essentially determined by the extent of the urothelial carcinoma, the metaplastic bone component could sometimes be increased considerably in size and thus required imaging procedures for follow-up. These included computed tomography (CT), magnetic resonance imaging (MRI) and positron emission tomography computed tomography (PET-CT). This has been illustrated in a case of retroperitoneal metastasis reported by Boudabbous et al., through which CT has been demonstrated as the best imaging modality that allows follow-up of lesion and detection of new lesions. CT imaging feature consists of hyperdense mass resembling cortical bone that shows moderate hypermetabolism (maximum SUV, 5.1) on PET – CT and appears hypointense on all sequences with peripheral enhancement on MRI.

The mechanism and the pathogenesis of metaplastic bone have not been completely elucidated [15]. However, we can firstly note that the urothelial carcinoma was of high grade (Grade 3) in the great majority of the 20 cases described with SOM, including our case. Grade 2 and grade 1 have been only reported respectively in 4 cases [2, 4, 6] and 2 cases [1, 11]. Tumor grade was not available in the last 4 cases [8, 13]. A possible relationship between the tumor grade and the bone forming capacity of urothelial cells could be evocated, suggesting that this capacity would be enhanced when the grade increased. Moreover, some authors suggested a possible correlation between the bladder epithelium and the local concentration of calcium, inorganic phosphate as well as the alkaline-phosphatase activity [1]. They revealed through several experiments the ability of urinary epithelium to induce osseous metaplasia. This is not due to its cell specificity but to its chemical and enzymatic products and also to its property to keep local enzymatic concentration constantly high, the main condition to obtain metaplastic bone [1, 3]. Another hypothesis suggested a transformation of stromal mesenchymal cells into osteoblasts which could be induced by bone morphogenetic proteins (BMP). These last correspond to multi-functional growth factors that belong to the transforming growth factor β (TGFβ) superfamily which play especially an important role in new bone formation. In particular BMP 2 and BMP 1b are considered as active inducers of osteoblastic metaplasia respectively in primary and metastatic tumor sites [1, 3]. The fact that primary tumors in reported cases were preceded by surgery or TURB suggests the presence of scar tissue and fibrous change in which there would be an increased alkaline-phosphatase activity and fibroblasts would be more susceptible to bone evoking agents, particularly to BMP leading to bone formation. This hypothesis could be also considered in our case toward the history of radical prostatectomy and adjuvant radiotherapy. In addition, annual leuprorelin injection which is a luteinizing hormone releasing hormone agonist (LHRHa) plays an important role in normal skeleton homeostasis [16]. Leuprorelin therapy would in fact decrease bone mineral density and increase local concentration of alkaline phosphatase that could contribute to metaplastic bone formation [16, 17].

Some authors have also attempted to suggest that extracellular mucin deposition contributes to maintain an alkaline zone which have been found necessary for optimum activity of alkaline phosphatase. Furthermore, other factors of osteogenesis that encompass specific transcription factors such as Oct ¾, Sox2, c-Myc and Klf4 have been described. These lasts have been found to generate pluripotent stem cells. Under the influence of these similar transcription factors, fibroblasts that are present within the stromal component could differentiate into osteoblasts [18]. The pathogenesis of SOM associated with non-urothelial bladder carcinoma could be thereby correlated to bone inducing properties of extracellular mucin and specific transcription factors, as well as the bone evoking activity of the urothelium.

Therefore, 3 conditions would be necessary for bone formation which include osteogenic precursor cells, inducing agents and a permissive environment. A genetic predisposition may also be implicated and could explain the only pediatric case of SOM within a high grade papillary urothelial carcinoma despite the absence of particular triggering factors.

More interestingly, all those hypotheses could be comforted by the fact that SOM were also encountered in metastatic bladder tumor that involved regional lymph nodes, bones and tissue in close proximity, i.e. supraclavicular region, abdominal wall and retroperitoneal space within the context of previous surgery. As metastatic sites occurred in bone or proximities as well as in tissue scar, this would suggest a high local concentration of bone-evoking agents which favors SOM.

Osseous metaplasia should be recognized as a nonneoplastic change that could be encountered in benign (inflammatory lesions, benign tumors) and malignant conditions as seen in our present case, given that urothelial cells are well-known as bone-inducing agent [1, 2]. Microscopically, benign metaplastic bone usually consists of irregular, mature and lamellated bone trabeculae, outlined by osteoblasts, osteocytes and eventual osteoclasts. Those cells do not demonstrate atypia and are relatively distinct from the mesenchymal cells of the adjacent stroma. Osteoid deposits are also found and often areas of hemorrhage are present [19]. The routine use of immunohistochemistry is not mandatory to assess the diagnosis as morphological features are usually well-defined. This could explain the absence of immunohistochemical (IHC) analysis for SOM in all those cases described. However, specific antibody stains could be performed. Osteoblasts stain positive for alkaline phosphatase, CD56 and SATB2. Expression pattern of osteonectin and osteocalcin could be found on osteoblasts, as well as on osteocytes and the surrounding matrix. Osteoclasts demonstrate positive immunoreactivity for Tartrate-resistant acid phosphatase (TRAP) and CD68. Special stains such as von Kossa, Masson’s and Goldner’s Trichrome could be also used to differentiate osteoid deposits [20].

The main histological differential diagnoses that should be distinguished from SOM in urothelial bladder carcinoma are sarcomatoid carcinomas (SC) of the bladder which constitute an important challenge for they definitely change the course and the treatment of the case [1, 4]. In the last World Health Organization classification of urinary tract tumors, these lesions are defined as biphasic neoplasms composed of an admixture of malignant epithelial and mesenchymal elements [21, 22]. These lasts could demonstrate osteosarcomatous differentiation, usually formed by urothelial carcinoma coexisting with osteosarcoma in which bone formation is an element of the mesenchymal malignant component. This variant of SC should be firstly eliminated, even though the incidence is rare. The mesenchymal component is characterized by high grade sarcoma with spindle, oval or round tumor cells of variable size showing marked nuclear pleomorphism. Bizarre giant cells and osteoclast-like multinucleated giant cells are not uncommon. Those cells are usually seen within abundant lace-like osteoid that could be associated with chondroid matrix. MDM2 and CDK4 immunostaining are not specific for diagnosis but could be expressed by tumor cells. Osteoid matrix stains positive for vimentine and alkaline phosphatase and positive immunostaining for S-100 protein could be identified in chondroid areas. Ki 67 and p53 are not specific but could be considered as indicators of malignant potential. They are highly expressed in osteosarcoma and usually negative or mildly expressed in metaplastic conditions. This histological variant of urothelial carcinoma has a poor prognosis. The treatment consists of radical cystectomy [15, 23]. Tumor cells are occasionally radio-chemosensitives. Most patients die within 6 months due to local spread with urinary tract obstruction and secondary infection [23].

Noteworthily, in this context of SC, urothelial carcinoma could also coexist with leiomyosarcoma, chondrosarcoma, rhabdomyosarcoma, rarely liposarcoma, and more than one type of heterologous differentiation may be present [15]. Those components are obviously morphologically different from metaplastic bone and thus, should not be specifically considered as diagnoses problems.

In the context of extensive metaplastic bone as well as metastatic urothelial carcinoma associated with SOM, differential diagnosis of bone formation should be also established on imaging. Radiation-induced osteosarcoma should be firstly suspected in patients who have undergone radiotherapy, as far as lesions involved the irradiation field. A latency of 6 months from the exposure is sufficient to affirm the diagnosis. Secondly, extraskeletal osteosarcoma should also be evocated. Patients are mostly adults, aged over 40 years and the prognosis is almost always poor. The typical CT presentation of osteosarcoma consists of heterogeneous areas with focal hypoattenuation that corresponds to necrosis or hemorrhage. On MRI, these tumors usually demonstrate intermediate signal on T1 with enhancement or low signal both in T1 and T2 sequences. Sarcomatoid carcinoma, recognized as the main differential diagnosis of urothelial bladder carcinoma with SOM, should be also suspected on imaging, especially on CT and consists of a large heterogeneous mass with hyperdense components.

The presence of metaplastic bone within bladder urothelial carcinoma does not change the therapeutic process that essentially differs according to tumor stage and depends on muscular invasion [24, 25].

Of the 12 cases of primitive tumors reported with SOM, 4 cases had favorable outcomes [1, 5, 6, 9]. In these cases, patients were correctly treated using procedures that are based on consensus guidelines according to tumor stages. Three other cases had negative outcomes characterized by patient death [4, 8] or persistent lesion on follow-up [2]. Death is usually due to metastasis or unresectable tumors with or without associated complications such as pyelonephritis [2, 4, 8]. In the 5 remaining cases, outcomes were not available [1, 2, 4, 7, 10]. Concerning the 7 cases of metastatic urothelial carcinoma reported in the literature, only one case [3] had available outcomes, marked with disease progression mainly resistant to radiotherapy.

We could notice through those observed clinical courses, including the present case, that the disease outcomes are based on treatment, particularly on its response or effectiveness as well as on its clinical feasibility. The quality of follow-up may also have a major impact on outcomes and survival.

## Conclusion

Stromal osseous metaplasia is a benign condition rarely found within urothelial bladder carcinoma. Detailed mechanisms underlying this phenomenon have not been completely elucidated and need further investigations. The presence of this feature does not change the treatment approach of urothelial carcinoma which is related to histologic grade and tumor stage. This last is relatively based on muscle invasion. However, metaplastic bone should be differentiated from malignant osteogenous conditions. Sarcomatoid carcinoma constitutes the main differential diagnosis that obviously changes the treatment approach and thus represents a challenging problem. The prognosis is poor in all cases of sarcomatoid carcinoma and outcomes are characterized by a high incidence of recurrences. Therefore, a long-term follow-up is recommended.

## Data Availability

All data generated or analyzed during this study are included in this published article.

## Abbreviations

BCG: Bacillus Calmette-Guerin
BMP: Bone morphogenetic proteins
CT: Computed tomography
IHC analysis: Immunohistochemical analysis
LHRHa: Luteinizing hormone releasing hormone agonist
MRI: Magnetic resonance imaging
PET-CT: Positron emission tomography computed tomography
PSA: Prostate-specific-antigen
SC: Sarcomatoïd carcinoma
SOM: Stromal osseous metaplasia
TGFß: Transforming growth factor ß
TURB: Transurethral resection of bladder
